# Authors’ reply

**Published:** 2011

**Authors:** Prateek Agarwal, Mahesh Kumar, Vipul Arora

**Affiliations:** Department of Neuro-ophthalmology, Aravind Eye Hospital and Post Graduate Institute of Ophthalmology, Tamil Nadu, India

Dear Editor,

We are thankful to Nithyanandam[1] for keenly persuing our article.[2] We did not investigated all our patients straight away with magnetic resonance imaging (MRI) because of cost constraints and other causes of disc edema which could be excluded initially with computed tomography (CT) scan alone. After excluding all other causes of disc edema by CT scan, the patients with presumed idiopathic intracranial hypertension (IIH) (308) were evaluated with MRI and magnetic resonance venography (MRV). MRV was performed only when MRI was not supportive in diagnosis of cerebral venous sinus thrombosis (CVST), or there was strong clinical suspicion of CVST due to associated risk factors, namely, male sex, nonobesity, postsurgery, use of oral contraceptives, deep vein thrombosis, and hypercoagulable states in the presence of normal MRI.

The main aim of the study was to report the rate of occurrence of CVST, highlighting the role of MRI and MRV in patients with presumed IIH. The visual loss in the cohort was not analyzed. We agree that all patients with CVT require regular monitoring of visual function, both central acuity and visual field analysis, to prevent/reduce irreversible visual loss.
